# Augmented Hearing of Auditory Safety Cues for Construction Workers: A Systematic Literature Review

**DOI:** 10.3390/s22239135

**Published:** 2022-11-24

**Authors:** Khang Dang, Kehinde Elelu, Tuyen Le, Chau Le

**Affiliations:** 1 Department of Informatics, Ying Wu College of Computing, New Jersey Institute of Technology, University Heights, Newark, NJ 07102, USA; kdangho@g.clemson.edu; 2 Glenn Department of Civil Engineering, College of Engineering, Computing and Applied Sciences, Clemson University, Lowry Hall, Clemson, SC 29634, USA; kelelu@g.clemson.edu; 3 Department of Civil, Construction and Environmental Engineering, College of Engineering, North Dakota State University, Fargo, ND 58102, USA; chau.le@ndsu.edu

**Keywords:** Artificial Intelligence (AI), auditory signal processing, hazard detection, auditory situational awareness, construction safety

## Abstract

Safety-critical sounds at job sites play an essential role in construction safety, but hearing capability is often declined due to the use of hearing protection and the complicated nature of construction noise. Thus, preserving or augmenting the auditory situational awareness of construction workers has become a critical need. To enable further advances in this area, it is necessary to synthesize the state-of-the-art auditory signal processing techniques and their implications for auditory situational awareness (ASA) and to identify future research needs. This paper presents a critical review of recent publications on acoustic signal processing techniques and suggests research gaps that merit further research for fully embracing construction workers’ ASA of hazardous situations in construction. The results from the content analysis show that research on ASA in the context of construction safety is still in its early stage, with inadequate AI-based sound sensing methods available. Little research has been undertaken to augment individual construction workers in recognizing important signals that may be blocked or mixed with complex ambient noise. Further research on auditory situational awareness technology is needed to support detecting and separating important acoustic safety cues from complex ambient sounds. More work is also needed to incorporate context information into sound-based hazard detection and to investigate human factors affecting the collaboration between workers and AI assistants in sensing the safety cues of hazards.

## 1. Introduction

The recognition of auditory safety cues at job sites plays a vital role in preventing injuries and deaths for construction workers [1]. Research has found a significant correlation between a lack of auditory signal awareness and an increase in unsafe actions leading to fatalities due to the failure to stay vigilant in hazardous situations [2,3]. Inadequate auditory situation awareness is often caused by declining hearing capability due to the use of hearing protection and the complex nature of construction noise [3]. Therefore, the development of wearable devices capable of automated detection of acoustic safety signals has received increasing attention from the research community.

Despite the critical need for augmenting the hearing of safety cues for construction workers, research in this area is still lagging. Previous studies on wearable safety devices have been focused on employing real-time data analytics to continuously measure a wide variety of safety performance metrics [4] other than auditory signals. Typical functions of existing wearable safety devices include physiological monitoring, environmental sensing, proximity detection, and location tracking of construction hazards [5]. Adopting computer vision algorithms to detect hazards is among the most popular approaches for extracting information from images or videos [6,7,8]. This approach still involves various challenges, such as limited field of view, illuminations, and occlusions of digital cameras that could harm the widespread use of this method at complicated construction sites. Others employed kinematic sensors, such as gyroscopes and accelerometers, to record the kinematic signals of the equipment and to detect its activities [9,10]. However, using kinematic sensors fails to detect hazards in such situations when sensors cannot be directly attached to the body of the machine that vibrates during operation, such as jackhammers, concrete pumps, and concrete truck mixers. It is also expensive and complex to deploy sensing devices on every piece of construction equipment. 

Audio-based hazard detection has emerged as a supplement technique that can augment workers in effectively monitoring the work environment. Processing audio signals for automated detection has meaningful implications because hazardous situations usually cause strong acoustic emissions. A variety of hazard detection models employing advanced machine learning techniques for signal processing have been developed in many other workplace environments other than construction sites [11,12,13,14,15]. They possess great potential to enable workers to quickly detect auditory safety cues that are important for their safety. These computing advances have been implemented in many different environments, including indoor and public environments [16,17,18,19,20,21,22,23,24,25,26], medical and health care systems [27,28], and working environments [29,30,31,32,33]. However, current research does not sufficiently address automated auditory surveillance for construction safety. 

The past review papers on auditory surveillance in construction were only focused on analyzing existing methods to support the monitoring of work tasks [34] and understanding workers’ use of wearable technology to prevent hearing loss [4]. This paper is the first attempt aiming to systematically review advanced Artificial Intelligence (AI) techniques in auditory signal processing and to provide insights into their potential capabilities for enhancing construction safety. The paper examines how hazardous situations can be detected by leveraging recent advances in audio signal processing and to what extent its efficiency and effectiveness can be improved. At the same time, it analyzes research and application trends and then identifies research gaps that require further research. The scope of this review includes academic articles on the significance of auditory situational awareness for construction safety and the applications of auditory surveillance in detecting hazardous situations. Moreover, a critical review of computational advances is summarized for researchers and practitioners interested in auditory signal processing.

## 2. Research Background

Many construction hazards produce strong acoustic emissions; thus, preserving or even improving the auditory situational awareness of construction workers could reduce the risk of fatal accidents on construction sites. This paper argues that auditory situational awareness is critical to the safety of construction workers because hearing safety-critical acoustic signals can allow timely responses to hazards. In this section, various types of construction accidents and their association with safety signals are first given. Then, the role of auditory situational awareness for construction safety is presented, followed by a critical analysis of the current state-of-the-art work on auditory surveillance in workplaces, including construction sites.

### 2.1. Construction Hazards

**Figure 1:**
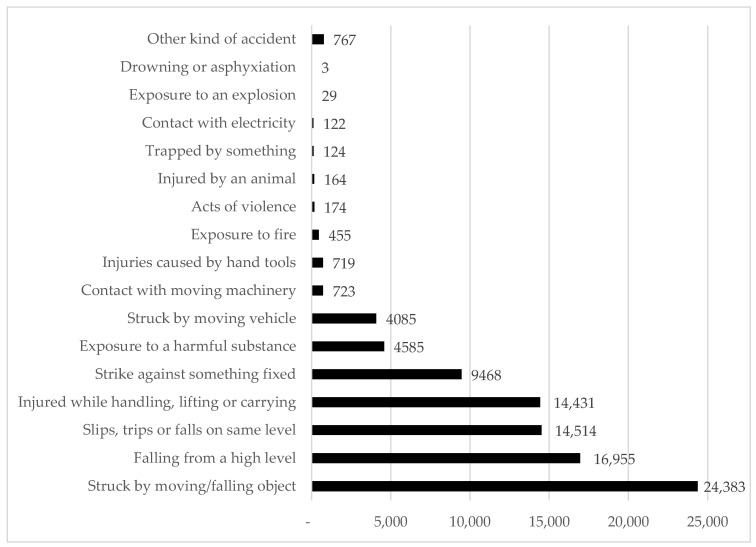
Total number of construction accidents by type in one year combined in the US, UK, and Israel [35].

Figure 1 presents common causes of construction accidents along with the number of cases in a one-year period combined in three countries, including the US, Israel, and the UK, recorded in 2008, 2011, and 2009, respectively [35]. As shown in the figure, it is apparent that “Struck by moving/falling object” is the most common cause associated with more than 24,000 incidents, followed by “Falling from a high level” with nearly 17,000 accidents. Furthermore, there were approximately 14,500 accidents due to “Slips, trips, or falls on the same level” and “Injured while handling, lifting, or carrying”. Other types that also cause a significant number of accidents are “Strike against something fixed”, “Exposure to a harmful substance”, and “Struck by a moving vehicle”. 

Another report by [36] revealed the number of fatal cases from the Occupational Safety and Health Administration (OSHA) between 1990 and 2007 for different types of construction hazards, including (a) getting struck by equipment or vehicle, (b) getting struck by part of equipment/material, (c) equipment rollover, (d) equipment in water, and (e) electrocution in contact with the equipment. Accordingly, struck by equipment/vehicle hazard was the cause of 659 cases, accounting for 87.7% of the total fatalities. In addition to struck by moving equipment, workers were also involved in accidents by the hit of equipment buckets, material being dropped or lowered during transferring, electrocutions when workers were touching equipment that contacted power lines, rollovers when the equipment was operated on steep slopes, and drowning when equipment rolled into deep water. 

As noted above, equipment is one primary source of construction hazards. Table 1, adopted from Ref. [36], ranks 16 types of construction equipment by their frequencies of job-site fatal accidents. Overall, dump trucks had the highest frequency of involvement in fatality cases with 173 incidents, almost two times higher than the combined fatalities cases by backhoe excavators (second rank) and private vehicles (third rank) with 50 and 42 incidents, respectively. Following the top three vehicles, dozers, graders, front-end loaders, and forklifts also had a relatively high quantity of incidents, ranging between 30 and 40 each. Another point that could be derived from the table is that most of the accidents occurred when the equipment was moving backward. For example, 91.1% of the fatal accidents caused by dump trucks were associated with reserve movement. This percentage for many other types of equipment, including graders, loaders, and water trucks, was reported to be over 90% and it was at least 50% for excavators, dozers, front-end loaders, forklifts, tractor-trailers, compactors, and scrapers. Those statistics of accidents indicate the great hazardousness of construction equipment in the reverse direction.

Furthermore, it is worth mentioning that only a small portion of those accidents due to the reverse direction occurred when the backup alarm was not operated. For instance, the alarm was not functional in only 12.1% of the total cases for dump trucks and even 4% for backhoes. Noticeably, 26.7% of scrapers’ cases were attributed to reverse alarm failures. This is probably due to large blind areas on all sides of the equipment. According to Schultheis’s [37] report, the blind area range for scrapers is high compared with other equipment types. A scraper operator typically cannot easily see what is behind, which decreases the visibility in a reverse direction. These data suggest that a reserve alarm was largely inefficient in preventing fatal accidents. This may be due to the habituation of warnings or the impact of other ambient noise. Thus, augmenting the worker’s auditory sensing would be an urgent need.

### 2.2. Significant Role of Auditory Situational Awareness for Construction Safety

**Figure 2:**
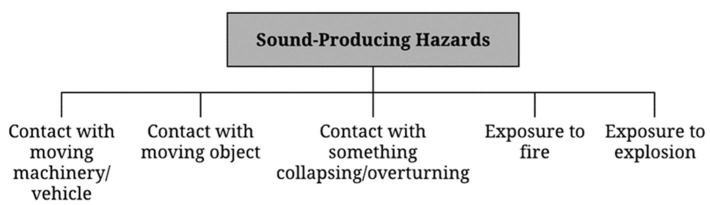
Construction hazards detected by acoustic signals.

Of those construction hazards discussed above, many are associated with acoustical signals. Early recognition of these safety-critical sounds could be beneficial in preventing construction accidents. Figure 2 classifies construction hazards that can be detected by their associated sounds. These sound-producing hazards are: contact collision with moving machinery/vehicles, objects, collapsing/overturning, exposure to fire, and exposure to an explosion. Moving machinery/vehicles, which can cause contact hazards, are mentioned in Table 1. For the type of accident in close contact with moving objects, the hazardous scenarios include missing footboards on a scaffold, moving a crane with a load where workers are present, working with loose materials (blocks) at a height, working with façade elements on a scaffold at a height, and working with unsecured hand tools at a height. Regarding being trapped by something collapsing/overturning, hazards come from improperly secured slab formwork and improperly supported wall formwork. The awareness of these signals would help construction workers evaluate the situations and make proper responses to any potentially hazardous situations

Auditory situational awareness is understood as a cognitive process of acoustic signal recognition. According to the cognitive model of construction workers’ unsafe behaviors (CM-CWUB) developed by [2], the process of responding to a potential hazard includes five stages: (a) obtaining information, (b) understanding information, (c) perceiving responses, and (d) selecting responses and taking action. The model is useful as it can allow us to understand the mechanism of human errors as well as how unsafe behaviors are made at various cognitive stages. In the context of auditory situational awareness in construction safety, workers need to receive the signal and recognize its original source and direction (e.g., engine sound from a heavy vehicle moving towards the workers). Based on their experience, judgment will be made regarding the occurrence of a certain potential hazard and the corresponding safety actions. It is worth noticing that the first stage (obtaining information) and the fourth stage (selecting responses) are the two critical sources of unsafe behaviors of construction workers. Unfortunately, the loudness level of sound produced in the construction site due to its complicated nature is loosely defined and regulated, making it hard for workers to identify hazards. In addition, making high-stakes decisions to deal with dangerous situations is prone to errors. Due to the above barriers, cognitive assistance is necessary to allow construction workers to timely detect possible hazardous auditory events. Augmented hearing devices would be particularly needed to assist workers in evaluating situations and providing decision-making recommendations.

The ASA of construction workers is affected by various factors that can be categorized into job-site-related and worker-related factors, as summarized in Table 2. Regarding the job-site-related factors, the most significant cause of the decrease in ASA is the complexity of ambient sounds. Due to loud and complicated noises, the audible sensing of workers to hazards becomes limited. This limitation prevents construction workers from perceiving their surroundings and recognizing alerts of impending dangers. It is reported that the performance in signal recognition of normal-hearing listeners decreases as the number of concurrent sound sources increases [38]. Using hearing protection to prevent hearing loss would make further difficulty in maintaining workers’ ASA. As a result, essential sounds can be easily ignored or misidentified [39]. 

Additionally, worker-related factors such as gender and occupational hearing loss are reported to significantly impact the worker’s hearing capability. Research suggests that men have a higher degree of hearing difficulty than women [38]. Hearing loss in men is more prevalent because they are typically tasked to work in places exposed to loud noise [40]. Another research on the causes of hearing loss, investigated by Rigters et al., [41], found that men tend to have more hearing loss due to high frequency sounds than women. This may attribute to the severe hearing issues among male workers in construction as construction equipment noises are typically sounds with a high frequency (above 2000 Hz) [42]. In addition, workers with normal hearing still encounter a high level of hearing difficulties if exposed to hazardous noise [3]. The study in [3] showed profound challenges relating to job-related conservation for noise-exposed and hearing-impaired workers. The awareness of these issues is crucial to the future development of appropriate protective measures for noise-exposed and hearing-impaired workers. The next section will introduce the methodology adopted to develop this qualitative literature review on using auditory surveillance for hazard detection in the construction industry.

## 3. Methodology

**Figure 3:**
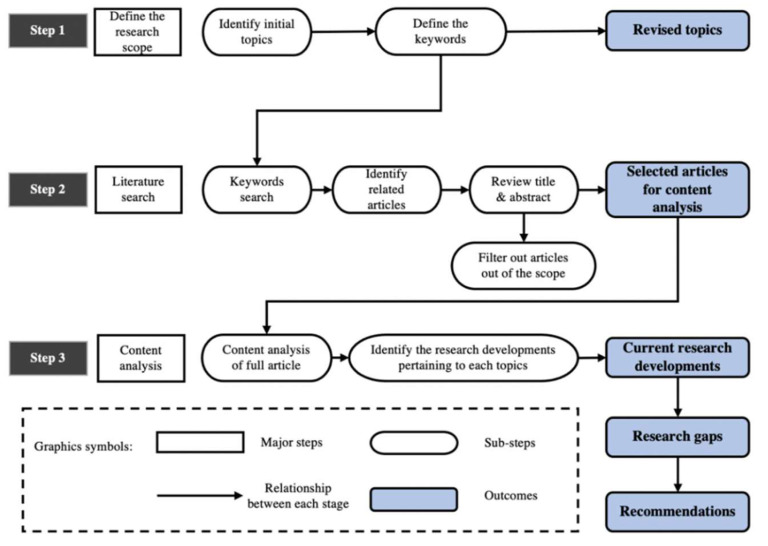
Research methods and procedure of this study.

This paper involves a literature review of academic articles that focus on the capabilities of advanced AI for auditory signal processing and sound surveillance in supporting construction safety. This research employed the review method proposed by [43], consisting of the following three main steps (see Figure 3): (1) defining the research scope based on identified keywords; (2) collecting the articles within the scope of this review by conducting a keyword search in the scientific databases; (3) content analysis to identify major research approaches, research gaps, and recommendations for future research. The details of each step are provided in the following sections.

### 3.1. Step 1: Define the Research Scope

**Figure 4:**
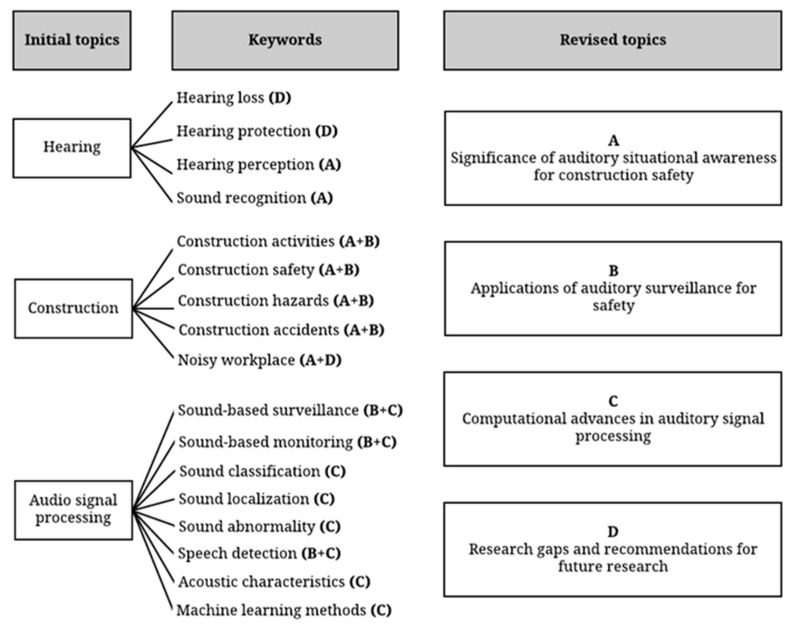
Identify the scope of this review based on keywords.

The work scope of automated auditory surveillance is broad and diverse. There are various sorts of acoustic events present in our everyday environment. For example, the most typical acoustic events in the office come from people chatting, doors opening and closing, and computers and other devices. At the same time, an automated auditory surveillance system could have many different purposes, including automated management of safety, security, and monitoring applications. To define the research scope, three main topics closely related to automated auditory surveillance for construction safety, referring to “hearing”, “construction”, and ”audio signal processing” (see Figure 4), were identified. For this study, articles with topics or abstracts containing either “construction safety” and “hazard” combined with any of the sentences, such as “sound activities”, “workers hearing”, “audio signal processing”, and “noise”, are studied. The research used for this study is not limited to studies conducted in the United States. Based on these initial topics, we found 17 related keywords previously used in past research, including four for hearing-related, five for construction-related, and eight for audio signal processing topics. After collecting relevant keywords, we revised the initial topics into more narrowed and specialized ones. It is worth noting that a keyword can relate to one or more revised topics.

### 3.2. Step 2: Literature Search

**Figure 5:**
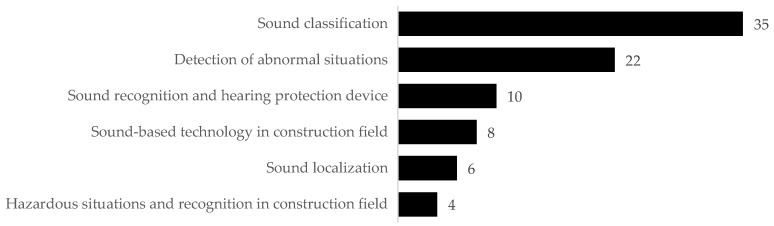
The number of reviewed articles by main contents.

In the literature search, all keywords shown in Figure 4 were used to search for related publications through the publicly available scientific databases, including Google Scholar, Web of Science, Elsevier, WILEY, ASCE, Springer, IEEE Xplore, and ACM Digital Library, published from 1998 to 2022. This process resulted in a total of 178 articles. Only the papers published in high-impact journals and conference proceedings were considered. After identifying the initial set of related publications, their title and abstracts were reviewed by filtering out the articles outside of our research scope through a content analysis thereby removing irrelevant documents. As a result of this process, 85 articles were included, which were then critically reviewed by the authors. The statistics of reviewed articles are shown in Figure 5. A total of 35 articles were found regarding the sound classification, while 22 were found for the detection of abnormal situations such as unusual sound produced by a piece of equipment. For sound recognition of workers with hearing and detecting device of surrounding situations 10 articles were found. Less than ten articles present the application of sound-based surveillance in the construction field. Only four articles study the detection of hazardous situations in the construction field and six articles were found for sound localization. Therefore, studies on the localization of sound sources to prevent hazards on the construction site are still in the early stages and are yet to be fully exploited.

**Figure 6:**
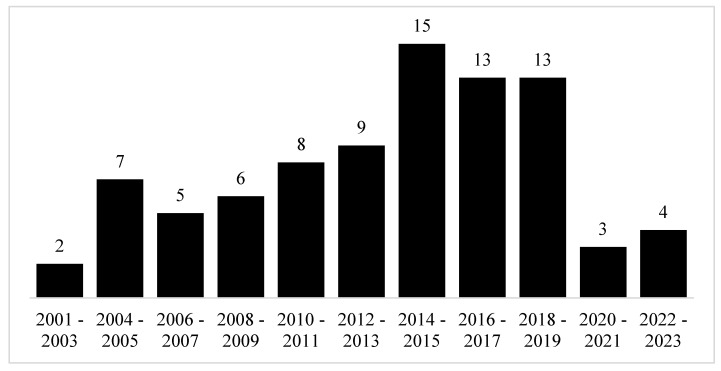
The number of reviewed articles by years.

To highlight the research trend on automated auditory surveillance, we summarized targeted articles every two years, as illustrated in Figure 6. The number of articles throughout the last two decades is increasing, indicating that more and more researchers are interested in sound-based surveillance. Especially within the last six years, the total number of articles from 2014 to 2019 reaches more than 40. This number proves that studies on the topic of automated auditory surveillance are crucial in today’s world.

### 3.3. Step 3: Content Analysis

In this step, the 85 identified articles found relevant to automated auditory surveillance for construction safety were rigorously examined. The articles were reviewed in the following aspects: (1) the role of auditory situational awareness in construction safety, (2) advanced methodologies in auditory signal processing, (3) applications of signal processing in safety surveillance, and (4) benefits and challenges. This step also involved critically analyzing the leading research gaps in previous studies. The findings of these steps are presented in the following sections. 

## 4. Results

### 4.1. Audio Signal Processing Applications and Methods

#### 4.1.1. Application Contexts of Auditory Surveillance

Auditory surveillance has been applied to detect abnormal events in various contexts. As shown in Table 3, the literature shows the implementations of sound-based technology in the following areas: home security [23], public environments [16,17,18,19,20,21,22,24,25,26], office [29], medical and health care facilities [27,28], and in industrial plants [31,32].

Public environments have received the most attention from the research community. Despite complicated noises in public, many studies achieved promising results for the surveillance of abnormal sounds such as glass breaking, screams, gunshots, and explosions. For example, Ref. [24] developed a technique for detecting shouting events in a real-life railway environment. Not only are audio events automatically detected, but the positions of the acoustic sources are also localized [17]. Audio events of gunshots could be detected based on a novelty detection approach, which offers a solution to detect abnormality in continuous audio recordings in public places [16]. Another similar architecture for acoustic surveillance of abnormal situations under different acoustic backgrounds was built to detect vocal reactions (screams, expressions of pain) and non-vocal atypical events associated with hazardous situations (gunshots and explosions) [19]. Models for the detection of abnormal acoustic events from normal background sound were also developed by several authors [20]. A few efforts have been made focusing on the detection of crimes in elevators [25] and the detection of human emotions based on verbal sounds in hazardous situations [18,26]. Ref. [29] built a system for acoustic surveillance to detect abnormal sounds from people talking, opening and closing doors, and using computers and other devices in an office. 

In addition, many studies have implemented signal processing for the surveillance of healthcare facilities. For example, a system to extract sound features for medical telesurvey was developed by [27]. It can classify detected sounds into normal and abnormal types. The system’s purpose is to detect severe accidents such as falls or faintings at any place in the living area, which is useful for the surveillance of the elderly, the convalescent, or pregnant women. Furthermore, since older adults living alone potentially get into trouble when they fall and are even unable to call for assistance, a framework that detects falls by using acoustic signals by analyzing environmental sounds was proposed [28]. 

In industrial sectors, current research on applications of sound-based detection has focused on detecting abnormal behaviors of the machine or the equipment. Acoustic-based fault diagnosis techniques of a three-phase induction motor are presented to see if the motor is in bad or good condition [31]. Many rotating electric motors can be diagnosed using acoustic signals; this can prevent unexpected failure and can improve the maintenance of electric motors. The advantages of the proposed acoustic-based fault diagnosis technique are its non-invasive technique, low cost, and instant measurement of acoustic signals. A novel optimization technique for the unsupervised anomaly detection of sound signals using an autoencoder (AE) is proposed [32]. The goal is to detect unknown sounds without training data to identify abnormality or failure in the operation of the stereolithography 3D printer, the air blower pump, and the water pump.

#### 4.1.2. Principles of Audio-Based Hazard Detection

**Figure 7:**
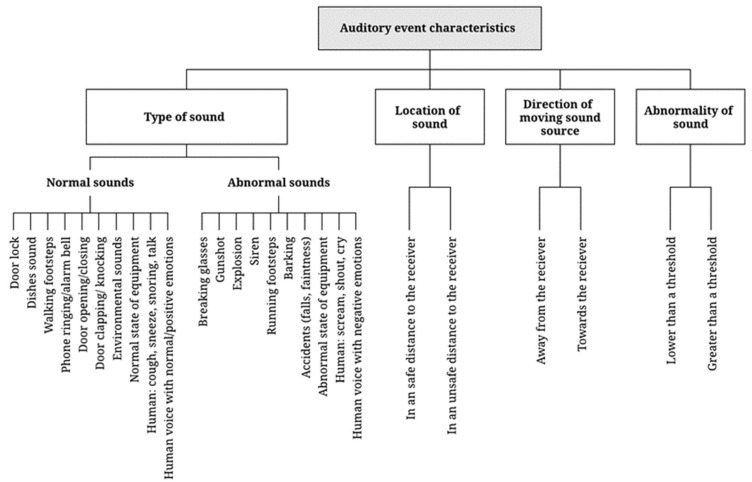
Indicators of hazardous events.

The detection of hazards using acoustic data is typically based on the extraction of the following four types of information: type of sound, location of the sound, the direction of moving sound sources, and the abnormality of sound (see Figure 7). These auditory event characteristics are used as input for detecting hazardous situations. The type of sound is the most common indicator used for detecting hazardous events and differentiating abnormal sounds from normal sound events. The occurrence of abnormal sounds, such as a gunshot or an explosion, is an important indicator of a dangerous situation requiring a quick safety response. Additionally, sound localization cues, such as the location and the direction of a moving sound source, are also essential for detecting potential abnormal events. They could inform receivers whether or not they are at an unsafe distance from the hazard. Moreover, measuring the abnormality of ambient sound has been used for evaluating hazardous situations, such as a scenario where a machine is operated and any abnormal sound that occurs in the parts or in the assembly process is often regarded as an abnormal sound and will require the attention of maintenance officers. Given the fact that we may not be able to classify all the unknown abnormal sound events that occur during equipment operational noise inspection, the anomaly would be useful in cases where we are developing the abnormal noise inspection device to automate the process.

**Figure 8:**
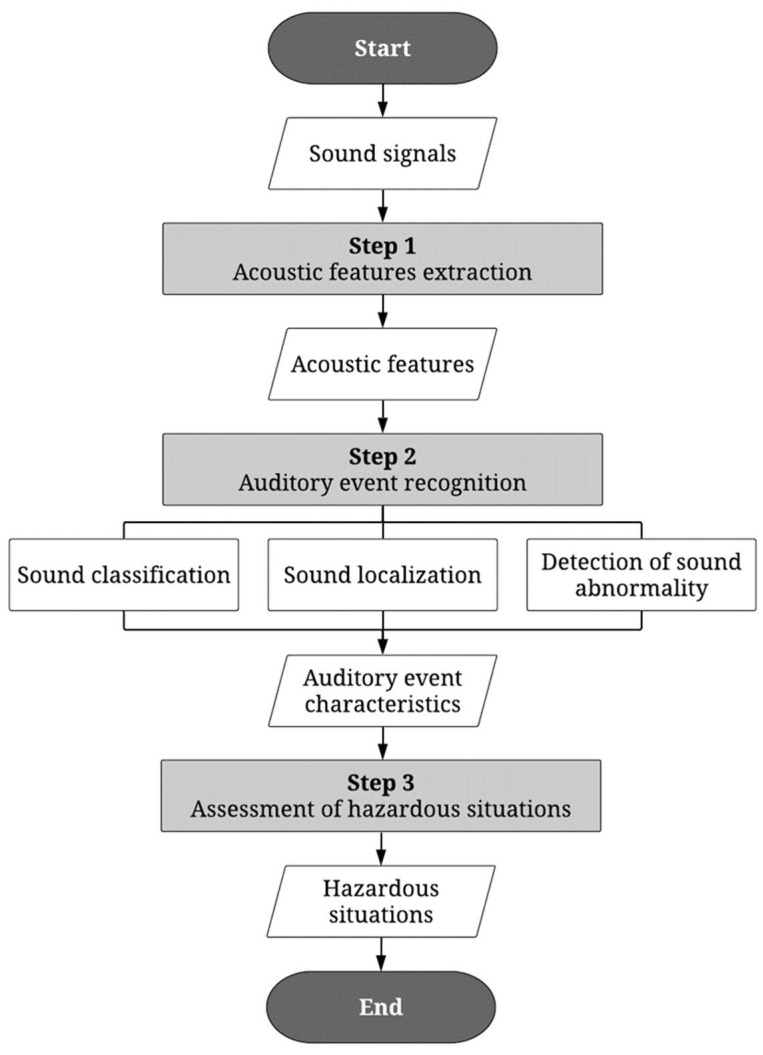
Overall architecture of auditory signal processing to detect hazardous situations.

Automatic detection of auditory cues requires computational advances in processing the auditory signals. As discussed earlier, hazardous situations are detected based on auditory features, including the type of sound, sound localization, and sound abnormality. Figure 8 provides a typical procedure of sound-based hazard detection found in various literature. The process entails three steps: (1) acoustic feature extraction, (2) auditory event recognition, and (3) assessment of hazardous situations. Of those steps, recognizing auditory events in Step 2 is the most critical step in the signal-processing procedure. This paper is therefore focused on the critical review of recent computational advances in sound classification, sound localization, and the detection of sound abnormality, which are required to recognize auditory events. Common types of acoustic features to be extracted from Step 1 will also be discussed.

#### 4.1.3. AI-Based Sound Classification

**Figure 9:**
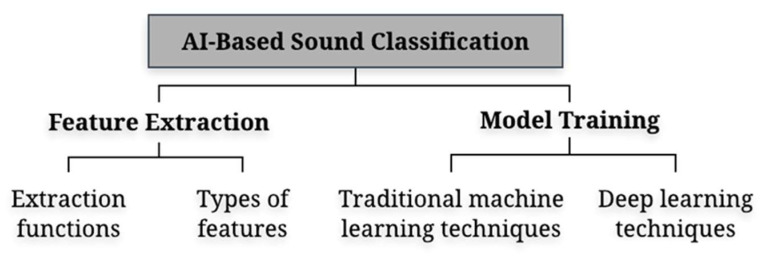
Main structure of AI-based sound classification.

This part focuses on the classification of sounds using AI. The main processes of AI-based sound classification are depicted in Figure 9. The first and foremost step is to extract the acoustic features from audio signals. Depending on the type of sound, a specific extraction function is used to extract the features. After all the necessary acoustic features are extracted, machine learning techniques, including traditional techniques and deep learning techniques, are employed to train models for sound classification. Common methods of feature extraction employing machine learning techniques are provided below. 

##### Feature Extraction

Feature extraction is an inevitable step because time-domain signals contain irrelevant data to directly use for sound classification. It is crucial to use appropriate features for successful classification as lousy features contain less discriminating power, which could hardly be classified. The summary of acoustic features used in sound classification is summarized in Table 4. Acoustic features can be grouped into cepstral features, spectral features, energy features, and temporal features.

**Figure 10:**
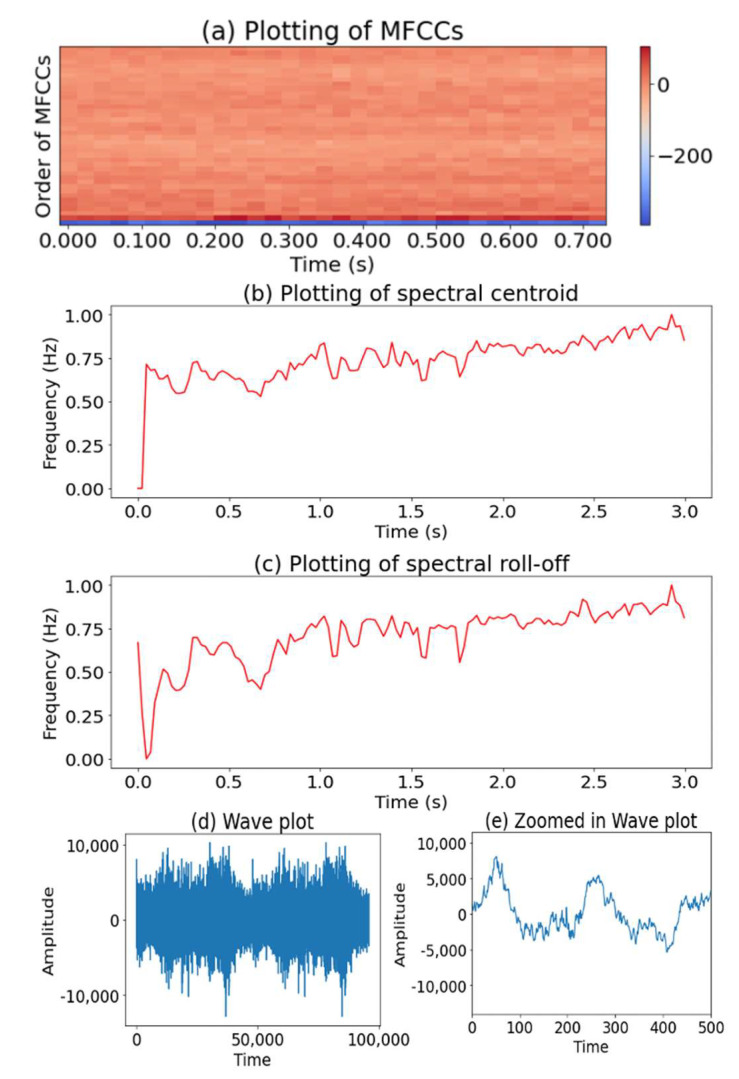
Graphic representation of acoustic features extracted from the sound of a moving grader.

Cepstral features are commonly used to define auditory signals. A cepstrum is computed by taking the Inverse Discrete Fourier Transform (IDFT) of the logarithm of the Fourier transform of a given signal. As shown in the table, there are various variations of cepstral features. Mel-Frequency Cepstral Coefficients (MFCCs) are the most popular cepstral features, which are specifically based on the power spectrum of sounds. An example of the MFCC’s representation extracted from the sound of a moving grader is shown in Figure 10a. While the positive values of MFCCs represent a sonorant sound with low-frequency signals, negative values represent a fricative sound with high-frequency signals. MFCCs have been used to extract the spectral envelope of signals in many audio and speech recognition applications [11,12,13,15,17,24,27,44,45,46,47,48,49,50,51,52,53,54,55,56,57,58,59,60,61,62,63,64,65,66,67]. Many researchers used only MFCC parameters [11,12,13,45,46,47,50,51,54,55,56,57,58,59,64,67], while a few others used MFCCs in conjunction with other spectral features including Zero Crossing Rate (ZCR), spectral roll-off, and spectral centroid [27]. 

In speech processing, Linear Prediction Coefficients (LPC) have been widely used for the cepstral analysis of sounds since they well approximate the characteristics of vocal sounds and give a good performance in distinguishing normal talk and excited shouting [68]. The advantage of LPC is the simplicity of the algorithm, because past signals are modulated as the weighted sum of previous values to reduce an error function [61]. LPC has a variation called the Linear Predictive Cepstral Coefficient (LPCC). In an investigation [63], LPCC is obtained using a direct recursion from the LPC. Compared with LPC, LPCC can better handle sounds with sudden changes or that involve complex noises [68]. The Log Frequency Cepstral Coefficient (LFCC) is another commonly used type of cepstral feature. It is typically extracted by calculating the frequency domain of sound signals using the logarithmic filter bank. It has been shown to outperform LPC in demarcating between the vocal and the non-vocal events [68]. To address the limitations of MFCC for signals having strong temporal domain signatures, such as non-speech audio signals at low frequencies, Ref. [61] proposed another biologically feature, namely the Gammatone Frequency Cepstral Coefficient (GFCC). The uniqueness of GFCC is the use of a Gammatone filter bank, which is inspired by the human auditory filter response. A few attempts that use concurrently different cepstral and perceptual features have been undertaken when developing models for sound classification [62]. Rouas, Louradour, and Ambellouis [24] examined this approach by integrating MFCC, LPC, and PLP. The most effective feature sets from their study are the combination of MFCC and PLP. Janjua et al. [61] used the combination of MFCC, LPC, and GFCC to detect a rare event and to achieve precision and recall measures above 90%. Atrey, Maddage, and Kankanhalli [68] optimized the parameters for LPC, LPCC, and LFCC to detect various kinds of acoustic events.

Sounds can also be characterized using spectral features that are based on the frequency domain. Spectral features are the most generic features that can be applied to a wide range of signals. One example of a spectral feature is the spectral centroid, which indicates the location of the “center of mass” [27,44,62,63,65,66]. Spectral roll-off provides the same information as spectral centroid, but it is used to measure the frequency delimiting 95% of the power spectrum [17,27,44,63,65,66]. Figure 10b,c visualize an auditory signal’s spectral centroid and roll-off values. Spectral flux is another spectral measure that computes how quickly the power spectrum of a signal changes over time [44,63,65,66]. Mel spectrum represents the approximately logarithmic frequency sensitivity of human hearing. It also reduces the dimensionality of the spectrum for automatic sound classification in many applications [48,49,52,53,60]. Several other spectral features have also been used in previous studies, including spectral moments [17], spectral slope [17], spectral decrease [17], modulation spectral features [69], and spectrogram [70].

**Table 4:** Summary of acoustic features used in sound classification.

Acoustic Features	Features Set	References
Cepstral features	Mel-Frequency Cepstral Coefficients (MFCC)	[11,12,13,15,17,24,27,44,45,46,47,48,49,50,51,52,53,54,55,56,57,58,59,60,61,62,63,64,65,66,67]
	Linear Prediction Coefficients (LPC)	[24,61,63,68]
	Linear Predictive Cepstral Coefficients (LPCC)	[63,68]
	Log Frequency Cepstral Coefficients (LFCC)	[68]
	Gammatone Frequency Cepstral Coefficients (GFCC)	[61]
	Perceptual Linear Prediction Coefficients (PLP)	[24]
Spectral features	Spectral centroid	[27,44,62,63,65,66]
	Spectral roll-off	[17,27,44,63,65,66]
	Spectral flux	[44,63,65,66]
	Mel spectrum	[48,49,52,53,60]
	Spectral moments	[17]
	Spectral slope	[17]
	Spectral decrease	[17]
	Modulation spectral features	[70]
	Spectrogram	[71]
Energy features	Short Time Energy (STE)	[44,62,63,65,66]
	Discrete Wavelet Transform Coefficients (DWTC)	[27]
Temporal feature	Zero Crossing Rate (ZCR)	[15,17,27,44,62,63,65,66,68]

Other types of features used for signal processing include energy features and temporal features. Short Time Energy, which represents the energy of the short speech segment, is the most popular type of energy feature used for signal processing [44,62,63,65,66]. Short Time Energy can be represented using a wave plot, as illustrated in Figure 10d. The plot shows the loudness of the sounds along with a given time range. Another commonly used energy feature is the Wavelet-Based Coefficient (DWTC). It is computed through the wavelet transform function. Models with DWTC could diminish processing time, due to the use of fewer parameters, but showed lower performance than models with MFCC [27]. Zero Crossing Rate (ZCR) is the most popular temporal feature in speech recognition and sound retrieval [15,17,27,44,62,63,65,66,68]. It indicates the number of times when the signal amplitude crosses the zero value [27]. It is more popular for use in discriminating periodic signals from signals corrupted by noises. Figure 10e depicts the zoomed-in wave plot of 200 array columns extracted from the audio of a moving grader with two zero crossings.

Energy, temporal, and spectral features can be used simultaneously for sound classification. Valenzise et al. [17] used many acoustic features that are not too sensitive to SNR conditions, including ZCR, STE, spectral features, perceptual features, and periodicity descriptors. Moreover, they also used new features based on the auto-correlation function. Correlation features are comparable to spectral features, though they are computed starting from the auto-correlation function of each frame. The purpose of these features is to describe energy distribution over different time lags. In fact, much of the energy of impulsive noises, such as gunshots, is concentrated in the first time lag, whereas the energy of harmonic sounds, such as screams, is spread over a broader range of time lags.

##### Training of Sound Classification Models

**Figure 11:**
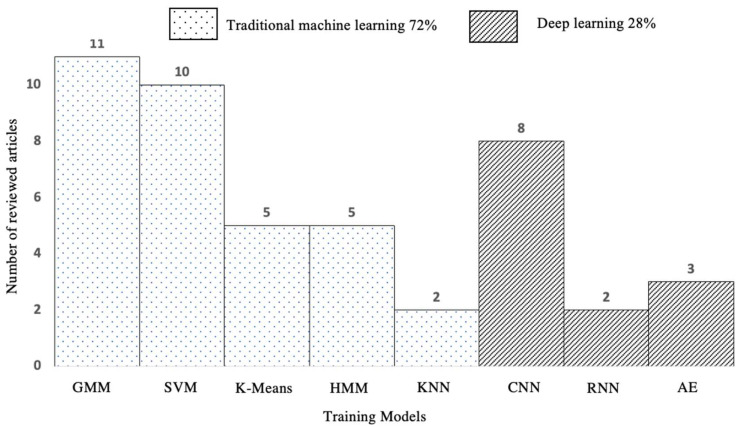
The number of reviewed articles by training models.

Sound classification is the process of analyzing acoustic signals to recognize and classify auditory events. Machine learning is the primary approach to classifying sounds found in most of the past literature. This section will summarize all the machine learning techniques used in the previous research for sound classification. As shown in Figure 11, machine learning techniques are grouped into conventional machine learning and deep learning. The traditional machine learning techniques include Gaussian Mixture Models (GMM), Support Vector Machine (SVM), K-Means Algorithms, Hidden Markov Models (HMM), and K-Nearest Neighbor Algorithms (KNN). Of those past techniques used in classifying sound, GMM was the most widely used method, with 11 reviewed articles. The deep learning approach comprises Convolutional Neural Network (CNN), Recurrent Neural Network (RNN), and Auto Encoder (AE). CNN was found as the most commonly used technique, with eight reviewed articles. 

Five different types of traditional machine-learning techniques for sound classification are listed in Table 5. GMM is a probabilistic model that fits the data points in a mixture of various finite Gaussian distributions with unknown parameters. There has been a large amount of research on the sound classification process using GMM due to its adaptability and simplicity. The sound classification GMM model developed by [16] for discriminating emotional contents and normal events showed that this method was significantly impacted by the noise in the training data. To reduce the false rates in the classification, Ref. [23] proposed adjusting GMM with an exponential criterion and a weighted least square solution. The classification performance also improved when GMM was modified using the Figueiredo and Jain algorithm [17].

Moreover, integrating a Bayesian criterion decision space with Gaussian distributions could well model the feature distributions of all the classes [24]. The use of two [25] or four [27] Gaussian components should be considered for building a classification model; although, higher numbers of components showed no improvement in the preliminary experiments conducted [27]. It is worth noting that adopting a more sophisticated multi-level classification approach will work better than single-level approaches [68]. Therefore, many studies considered GMM for classifying the hierarchical structure of sound events [19,20,71]. Finally, GMM could occasionally be used as a clustering method to generate sound data from functional representations by being trained on randomly sampled data from the training set [62].

While GMM captures the feature distributions of classes, an SVM technique aims to model a discriminative function to separate the classes [24]. It determines an optimal hyperplane that can separate the classes with minimized errors. In other words, it maximizes the margin between the decision boundary and the training samples to avoid overfitting on small training sets [21]. SVM works well for high-dimensional data and relatively small sets of labeled data points [74]. In addition, it is often applied in binary classification [28,57]. Using SVM, the selection of an appropriate kernel has a strong influence on the prediction performance. Several commonly used types of kernels are Radial Basis Function (RBF) kernel [11,30,57,72,74], linear kernel [44,74], and Gaussian kernel [73].

The K-Means algorithm, which searches for cluster centroids that minimize the Euclidean distance to the data points, was also used for auditory signal processing. This method does not require labeled samples to perform the clustering [21]; this is a great advantage over other methods for reducing the human labor necessary to train the event detection system on a new set of sounds. Thus, the K-Means algorithm is often used to perform clustering on auditory data [29,46,51]. Spherical K-Means is a modified form of the K-Means algorithm that is achieved by modifying the iterative update procedure for the K-Means algorithm [48].

Other popular traditional machine learning techniques are HMM and KNN. HMM relies on the statistical Markov model that trains a signal classification model by assuming the data to follow the Markov process. There are various applications employing HMM, such as the detection and classification system for sound surveillance [22], construction activity identification and task performance analyses [75], audio-based context recognition system [63], acoustic environment classification [45], and automatic detection of different types of acoustic events [47]. Many researchers proved it as a reliable classifier for sound classification [75]. The simplest machine-learning algorithm implemented for sound classification is probably KNN [57]. This method could properly classify linearly separable and non-linearly separable feature vectors. It can classify feature vectors using just a small number of training examples. Therefore, KNN is often used for signal processing and data classification of multi-class problems with a high recognition rate [31]. However, one noticeable drawback of KNN is time complexity, since, with a large dataset, the cost of calculating the distance between a new point and each existing point is very high, which in turn degrades the performance of the algorithm.

Recently, a Deep Neural Network (DNN) has been proven to outperform other traditional machine learning techniques in a variety of tasks for sound classification [58]. DNN is a complex feed-forward network comprising several hidden layers. DNN can produce a 54% higher performance than GMM in classifying audio concepts [59]. In another study that used DNN for detecting the typical sounds of everyday life, the classification performance was as high as 88% [58]. The use of DNN for recognizing acoustic events, such as footsteps, baby crying, motorcycle, and rain, achieved a classification accuracy of 60.3% compared with 54.8% for the conventional HMM classifier [60]. Recently, the following more complex architectures of DNN have been used for audio classification: CNN, RNN, and AE. Several studies have used Convolutional Neural Networks (CNN) with large input fields for sound classification of large audio datasets [12,15,55,62]. In a CNN network, each convolution layer generates a successively higher-level abstraction of the input and preserves its essential unique information. For sound processing applications, CNN is able to learn filters that are shifted in both time and frequency so that it can cover a large number of input fields [62]. The performance of CNN networks for signal classification is highly influenced by the optimum number of convolutional layers, which varies across different studies. Ref. [15] reported that CNN performed well with two convolutional layers, while three convolutional layers were suggested by [55]. Researchers also trained CNN models with a back-propagation mechanism consisting of pattern creation and testing processes [31]. CNN has shown impressive performance in auditory signal processing. Ref. [62] proposed to convert audio into vectors which are then trained using CNN. This approach achieved an average F_1_ score of 0.839. Table 6 sumarizes popular deep learning techniques for sound classification.

A few efforts have been made that use RNNs for sound classification. With RNNs, the information from previous time steps can circulate continuously inside the network through the directed cycles, where the hidden layers also act as memory [62]. Hence, RNN can capture better long temporal context information. A derivative of RNN is the Bi-Directional Long Short-Term Memory (BLSTM), which trains a special RNN by adopting a very long-ranged symmetric sequence context with a combination of nonlinear processing elements and linear feedback loops to store a long-range context. Although BLSTM has been used in a few applications to detect acoustic events with remarkable performance, its ability to convey contextual information in a long audio sequence was not really advantageous compared with CNN classifiers [76].

AE is a novel approach to acoustic novelty detection. When some of the input acoustic features are correlated, AE can learn those correlations and reconstruct the data from a compressed representation [76]. AE was reported to significantly exceed the performance of other traditional machine learning methods. The results from a study demonstrated that the AE approach outperforms GMM and HMM classifiers [77]. Furthermore, the use of an AE to minimize the reconstruction error of normal sounds could efficiently decrease the false positive rate [32]. 

#### 4.1.4. Sound Localization

Sound localization refers to the ability to identify acoustic sources in terms of direction and distance. It is one of the essential acoustic parameters that enables the ability to recognize and locate hazardous events. When dangerous objects or equipment are within an unsafe distance of a construction worker, detecting the location of acoustic sources can enable them to make appropriate preventive responses. Methods to localize sound events are mainly based on calculating the difference in the arrival times of the signal. Then, the similarity measure of the signal at different times is examined in either time or frequency domain for sound localization. For the time domain, the acoustic impulses reach the microphones at varying Times of Arrival (TOA) when they are spatially distant from one another. The signal’s Direction of Arrival (DOA) is determined from the recorded time delays using the given array geometry. Each pair of microphones in the array has a projected time delay. Then, using time delays and geometry, the best estimate of the DOA is determined, while the frequency domain is the difference between the time sound pressure reaching the array geometry and is mostly used to localize higher frequency sounds. Table 7 lists the methods for sound localization found in this review, of which the details are provided below.

Calculating the Time Difference of Arrivals (TDOA) of the signal is one approach to the detection of sound location. Valenzise et al. [17] adopted the Maximum-Likelihood Generalized Cross Correlation (GCC) method and linear-correction least-square localization algorithm for estimating the TDOA of the signal. Ref. [78] utilized the Euclidean Distance (EUD) to measure the similarity in the time domain. Other alternatives include measuring the similarity between signals at different arrivals based on the frequency domain. Compared with the time domain, the frequency domain showed significant improvements in the precision for sound localization as demonstrated by Satoh et al.’s [79] study, where they computed the cross-correlation in the frequency domain based on the Normalized Cross Correlation (NCC) method. Tarzia et al. [80] used Fast Fourier Transform (FFT) to measure the frequency domain similarity. Lastly, Wirz, Roggen, and Tröster [81] proposed an innovative method called the fingerprinting algorithm to measure the similarity based on the naive Bayes algorithm. Their results showed that the estimation of the quantitative distance in meters between a device D_A_ and another device D_B_ reaches an accuracy of approximately 80% when using ambient sound as a relevant source for obtaining proximity information.

Recently, a Deep Neural Network (DNN)-based approach has been proposed over the parametric approach (TDOA and IID) for sound source localization. Adavanne et al. [82] employed a convolutional RNN for sound localization of multiple overlapping sound events in three-dimensional (3D) space. The approach network takes a sequence of consecutive spectrogram timeframes as a multichannel input and maps it to a multi-output regression. The study shows that the proposed method is generic and applicable to any array structures and is robust to unseen DOA values, reverberation, and low SNR scenarios.

#### 4.1.5. Sound Abnormality Detection

Measuring sound abnormality is another challenging issue, given that preparing all labeled sound data related to hazardous situations is unrealistic. If a surveillance system is trained using the data of specific sounds, such as explosions or gunshots, it cannot be applied to detect other auditory events. A method for detecting unknown abnormal sound events is required to develop an automated auditory surveillance system that is useful in more general cases. To address this issue, Ref. [71] developed a method that models the abnormality of sounds without using any samples of labeled sounds. The technique can detect those sounds that rarely occur in a normal situation. It first processes sound in the usual situation and trains a statistical model of the normal sounds. After training the model, the system continues to process sounds and calculates the likelihood of the sound. If the likelihood value goes beyond a predefined threshold, that sound is considered abnormal. Lu et al. [83] used another approach to detect abnormal sounds using the case-based identification algorithm. In this method, it is necessary to first convert the sound data into feature representation vectors and then to apply an establishment distribution of a supervised learning model. This supervised learning requires a small training dataset of sample elements of abnormality. 

In the construction industry, sound equipment abnormality will affect the model performance since mobile equipment sound varies between different types of equipment. This could be because the acoustic characteristic of one sound is more difficult to train than that of another. Other equipment characteristics that may affect sound abnormality measurement are the models, brands, age, and maintenance programs. Obtaining different model metadata for the audio dataset would help us understand how they affect the detection capability. Collecting such data requires a significant effort in terms of time, cost, and human resources.

### 4.2. Audio-Based Surveillance in Construction 

#### 4.2.1. Feasibility of Implementing Audio-Based Hazard Detection in Construction 

The potential auditory indicators of hazards in construction are provided in Table 8. As shown, one of the most important types of sounds is the sound source of moving heavy equipment or machinery, which can cause collision hazards [30,33,36]. Furthermore, the detection of screams, shouts, or cry sounds is also supportive for acoustic surveillance and monitoring of negative situations, since human emotions are somehow delivered in sound events [17,18,19,20,23,24,25,26,27,28,61,62,68,76,77]. Other sounds released by people can also help detect abnormal situations. Detecting ground ambient sound (i.e., a group of people) allows workers to be aware of violent events, natural disasters, riots, or chaotic acts in crowds [26]. Some types of safety-critical sounds which lead to dangerous situations, such as alarms of fire, earthquakes, explosions, and gunshots, could improve the situational awareness of hazards [23,25,76,77]. For example, the detection of an explosion allows workers to stay away from the hazardous source [16] and gunshot detection allows workers to stay away from a gun attack [16,17,19,20,25,61,62].

Other than the classification of sound, sound source location [78,79,80,81], the direction of a moving sound source [78,79,80,81], and sound abnormality [71] are useful for detecting hazardous situations in the construction field. For example, the information on the location and the direction of moving construction equipment from the engine sound could help alert if a heavy construction vehicle is in proximity to a worker. Additionally, suppose the construction equipment breaks down by falling, collapsing, colliding, or by failures in the engine, the information of the abnormality in the sound could give a cue to carry out proper assistance, e.g., the noise produced when excessive vibration of equipment occurs that is not usually expected to vibrate. 

Existing frameworks for detecting safety cues solely rely upon a single type of information, for example, high-frequency filtering [84], sound identify classification [85,86], or direction of arrival of sound [87]. Using the information individually is insufficient for evaluating hazardousness in construction that requires a simultaneous consideration of many factors, including the size of the equipment in contact with, the breaking speed of a machine, the average reaction time of a worker, and the speed of the worker. Table 9 presents a list of hazardous situations in construction sites along with required auditory indicators. Specifically, hazardous situations include heavy equipment/machines being at an unsafe distance from workers when detected using the sound of moving equipment. The detection of equipment approaching or operating in an abnormal condition will be alerted if the direction of the moving sound source is toward the worker or if the sound source is abnormal. Other situations, such as someone crying/shouting, a crowd approaching, alert alarms, an explosion, or a gunshot, can indicate a hazardous situation. Defining hazardous situations that require quick and effective responses from construction workers is the priority of automated auditory surveillance in the construction field and could contribute to construction workers’ safety.

#### 4.2.2. State-of-the-Art Research in Auditory Signal Processing for Construction

There have been emerging studies on audio-based activity detection for improving construction management and productivity due to its advantages in terms of cost and applicability [88]. Other efforts have aimed to develop new methods for assisting workers in hearing critical sounds, which is a crucial need given the typical heterogeneity of sounds generated from diverse construction work activities, including static equipment and hand tools [89,90]. The examination of various OSHA accident reports by Hinze et al., 2011 [36] revealed that the heterogeneous nature of concurrent construction sounds (e.g., equipment sounds and alarms) indeed decreased workers’ safety awareness, since alarm signals may be drowned out or not audible enough for workers. They also reported that there were cases where multiple alarm signals issued warnings at the same time, influencing workers’ judgment and making the alert signals less effective or ineffective. However, due to the technical challenges of processing complex soundscapes, this problem has gained little attention from the academic community in the past decade. 

Only a few papers were found related to this technology in construction management. In general, previous studies were focused on the tracking of activities of construction equipment, identification of working and operation activities, proximity detection and alert systems, and embedded sensory systems. Of those areas, a majority number of studies aimed at monitoring the activities of heavy construction equipment to reduce operating costs [30,72,74,91]. This is probably because a large portion of the expenses in a construction project is allocated towards the operating costs of heavy equipment. For example, Ref. [92] applied the Hidden Markov Model (HMM) and a frequency-domain technique on spectrogram data to accurately classify types of construction sounds and to identify patterns from each type of construction task. The classified sound signals’ strength and location are visualized with a Building Information Modeling (BIM) platform. The acoustic signals from construction activities were used to calculate working periods to allow field managers to track work progress and productivity and to provide a means to efficiently enhance project schedule management. Ref. [93] worked on a sound monitoring system for the prevention of underground pipeline damage. To develop a dataset similar to what is found on the construction site, they collected working equipment sound data of typical construction threats, including excavators, hammers, road cutters, and electric hammers. They also collected the background noise of a typical construction environment, such as pedestrians, traffic, and wind sound. Two random forest-based classifiers were developed to detect suspicious sounds and to help prevent pipeline damages caused by construction activities. The endpoint was to create an alarm system that uploads a report if the duration of a construction threat sound exceeds the threshold value specified.

Another study proposed a hybrid system for recognizing multiple construction equipment activities [30]. The study trained a machinery task classifier on integrated data of both audio and kinematics using Support Vector Machines (SVM). The proposed system results indicate that a hybrid system is capable of providing up to 20% more accurate results compared with cases using individual sources of data such as images [6,7,8], sensors [9,10], and audio [75,91,94]. The system allows the construction managers to monitor and track productivity, equipment downtime/idle time detection, equipment cycle time estimation, and equipment fuel use control. Wei et al. [95] also developed a noise hazard prediction method that combines a wearable audio sensor with Building Information Modeling (BIM) data to predict and visualize spatial noise distribution on BIM models.

There has also been a widespread usage of machine learning algorithms to train sound data for construction activity monitoring. Ref. [75] implemented a supervised machine learning-based sound identification approach to enhance the monitoring of construction site activities. Ref. [89] also developed a risk assessment framework using a machine learning algorithm. This method used the activity classification information from auditory signals to estimate safety risks based on the occupational injury and illness information contained in historical construction accident data. All these audio-based frameworks for detecting construction activities are effective, especially for night-time tasks, since activities on construction sites can be detected regardless of visibility levels that are not suitable for image-based approaches.

Another line of effort on auditory surveillance in construction is focused on the identification of construction collision hazards. The advanced computational techniques in auditory signal processing for collision hazard detection in construction are motivated by strong acoustic emissions from equipment operation, since construction equipment often produces unique sound patterns while performing certain activities (e.g., moving vs. idling) [74], which can be used as an indicator of safety-critical cues or warning signals. For example, the research done by Lee and Yang [84] used a high-frequency sound (18 kHz, inaudible to construction workers) to analyze the doppler effect change caused by a single subject’s movements to prevent struck-by hazards. They installed a speaker on equipment that plays a predefined high-frequency sound. A smartphone carried by an on-foot worker was used to capture the sound produced by the speaker. The input signal was processed to extract the position of the equipment relative to the on-foot worker. The proposed technology was able to classify the movement direction and speed with 97.4% accuracy. Although the study proves its potential for detecting collision threats from equipment, the study still has some limitations. Firstly, they only tested a struck-by hazard situation involving a single type of moving construction equipment. Since the construction site is a complex environment in which multiple pieces of equipment work simultaneously leading to signal overlap, the mixture of similar sounds would prevent the recognition of movement of individual pieces of equipment. Another limitation was that the sound source was a speaker attached to the equipment, not the sound produced by the mobile equipment. This means the deployment will require expensive installation of sound speakers on every piece of equipment present on the job site. 

Recent studies by Refs. [85,86] developed sound classification models that can distinguish between mobile equipment and stationary equipment to support collision hazard detection. These studies collected and synthesized the sound of construction equipment at different signal-to-noise ratios and used the dataset to develop a machine learning model using a CNN for automated detection of mobile equipment occurrence. The efficacy of this model was tested on a real construction site and the result accuracy of the model was 99% in detecting sounds related to collision hazards when the signals were not buried in background noises. Compared with earlier efforts, Refs. [85,86] offer superior advancement, as their models are able to deal with complex soundscapes with overlapping sound sources, including mobile and stationary equipment and background noise (e.g., workers communicating, materials’ movement, and street noises). Another study that utilized equipment sounds for collision hazard assessment was performed by Ref. [87], which aimed at localizing the sound sources using the Direction of Arrival (DOA) signal processing techniques. The determination of sound location can supplement the sound classifiers developed by Refs. [85,86] to enable a more comprehensive assessment of the hazardousness-based situations. This information is vital for construction workers and safety engineers to precisely reduce false alarms by only notifying workers if they are in a danger zone based on the distance calculated using the direction of the hazard. For example, a mobile piece of equipment moving further away from an on-foot worker does not impose a hazard. 

Quantitative benchmarks between existing frameworks for preventing struck-by hazards are greatly difficult as they all were tested with nonidentical testing conditions (e.g., software, hardware, job-site characteristics, and assumptions). Therefore, some performance metrics, such as recall, cost, computational power, and data usage, can still be used for a fair comparison. In terms of performance comparison, they all yielded a competitive recall of 99% [85], 98% [96], 99% [85,86], and 100% [97]. Cost comparison was another metric to measure past success in preventing struck-by hazards with mobile equipment. Audio-based collision hazard detection by Ref. [85] requires little financial investment as the model can be quickly deployed on workers’ smartphones, while a relatively high deployment cost is needed for many sensor devices [97] or high-quality cameras [96,98].

## 5. Discussions on Future Research

Research on automated auditory surveillance has shown a rapid development momentum in the past decade. AI-based sound processing techniques show great potential for providing construction workers with powerful cognitive assistance to improve their auditory situational awareness of construction hazards. The most promising sound sensing frameworks, particularly for sound-based detection of construction hazards including those sound classifications, are those developed by Refs. [30,72,74,85,86,91]. Still, research on leveraging AI innovation to improve construction workers’ auditory situational awareness in construction safety involves various technological and practical gaps that require further research.

First, construction sites are a dynamic environment where many activities are performed concurrently, resulting in important signals being buried in complicated occurrences of overlapping soundscapes, including unwanted noises such as passing vehicles on highway projects. However, most previous studies used single sound sources in their training data [74,75,91,99]. It would be helpful to implement advanced sound separation models to separate noises from important signals prior to feeding them to the classification models. Another approach is to include overlapping sound samples in the training set. Ref. [85] was among very few efforts adopting this approach by training AI models on a large set of acoustic collision hazard signals mixed with noises at various sound-to-noise ratios. The study considered only stationary equipment sounds as background noise. Future research is needed to expand training sound mixture datasets to cover other background noises such as winds, traffic noise, and unwanted backup alarms. Additionally, the performance of sound classification models is required to be tested in diverse types of construction sites in terms of location (urban, suburban, and rural) and work type (highway, building, and offshore project). Construction sites are highly dynamic; machine learning models built on old data may be inapplicable for supporting the detection of new anomalies. Thus, continuously updating the model with new training data is extremely important for the success of these technologies. 

Another crucial gap in the literature is the lack of frameworks for detecting emergency sounds made by human subjects. Our review of recently published OSHA accident narrative revealed many types of audio cues requiring field personnel’s attention. Examples of verbal safety-critical signals mentioned in recent accident reports are “yelling”, “help”, “watch out”, “stop”, and “ahh”. The quote below is one great example illustrating the importance of preserving the audibility of such verbal signals.

“*At 2:00 p.m. on 13 February 2016, Employee #1 and Employee #2 were in an excavation installing sanitary sewer lines in a 13.5 foot trench near a gas line. A foreman saw that the excavation was caving-in and yelled at Employee #1 and Employee #2 to get out of the trench. Employee #2 got out of the trench without injury, but Employee #1 was not able to make it out and was killed at the scene*”

These sounds can be classified into pre-accident (i.e., “watch out”) and post-accident warning alerts (i.e., yelling for help). Both kinds of signals are crucial to enable co-workers to be aware of their emergency situations. Due to the exceeding noise level at construction job sites, it is extremely difficult for workers to recognize such signals. Unfortunately, there are no technologies available that are capable of improving workers’ awareness of such urgent sounds in noisy construction sites. Future research should explore the feasibility of AI in recognizing urgent speeches buried in unimportant noises, including normal speeches.

Sound classification alone is insufficient, given that the meaning of sounds in construction often depends on the context. The same type of sound may mean something different to different workers. For example, reverse alarms are an unwanted noise to those standing outside of the equipment backup trajectory. However, missing safety cues even in a short time can lead to deadly consequences. Thus, the preciseness in sound labeling (wanted vs. unwanted sound) is extremely crucial for construction safety. Unfortunately, none of the previous studies address this complexity of sounds in construction. Researchers would need to develop robust risk assessment models considering the job-site layout and workers’ spatial location and integrate them into sound classification models. Such integration will help reduce false alarms and enhance trust in the automation. The development of such AI models requires significant efforts to collect sound data representing a diverse range of real-life situations with regard to equipment types, equipment maintenance condition, and equipment movement trajectories relative to on-foot workers. It may be unrealistic to obtain field sound data for every type of construction project and activity, thus generating synthetic sound data using simulation platforms such as Pyroomacoustics may be necessary to supplement the data needs for deep learning models. 

Moreover, future efforts should be made to enable successful deployment of sound-processing AI models on wearable devices. There have been many wearable technologies developed for construction safety. However, most existing technologies are to support hazard sensing based on data that are different from audio signals. For example, Cho and Park [100] developed a tactile-based communication system to support equipment proximity detection. A similar tactile-based wearable device was recently developed and tested by Sakhakarmi et al., [101]. These experiments involved several shortcomings that need further validation. For example, human subjects were disallowed to perform their regular work activities during the test. This setting apparently did not reflect the nature of construction sites. Since workers are expected to continue working without distraction, future wearable devices must be comfortable and easy to use while performing other tasks. In addition, previous wearable safety devices in construction were built using a predefined type of feedback such as either text messages or visual alerts on a built-in screen. No studies have been found that investigate effective feedback mechanisms considering the complex nature of construction workflows. Research is needed to understand approaches suitable for effectively conveying the hazard detection outputs. Lessons learned from other fields, including hearing aids for hard-of-hearing people, would be a great point of departure. Refs. [102,103] are two examples of most recent work that compared different feedback methods, e.g., haptics, texts, and visuals, on various wearable devices including smart watches and brackets.

To ensure a high adoption of hearing aid devices for construction workers, future research also needs to examine the practical challenges and potential strategies to deal with technology adoption resistance. Factors such as those in Table 10 need to be considered to ensure successful adoption. Specifically, to increase the percentage of using wearable augmented hearing devices, effective interventions based on the protection motivation theory that aim to improve perceptions of severity, vulnerability, perceived cost, self-efficacy, and perceived response efficacy should be considered [104]. Once an augmented hearing model is integrated with hearing protection devices, the development of such technology should also take into account other factors, as identified by Ref. [105]. One of the factors is that the use of wearable devices among construction workers may be affected by social influence. Interventions considering personal and environmental factors using ecological and ergonomic models are required to promote adoption among construction workers. In addition, hearing-related problems such as noise-induced hearing loss or left and right ear differences need to be addressed [106]. Furthermore, the perception of noise and the perceived value of hearing assistance are essential factors that affect the use of augmented hearing devices. Research shows that most construction workers still refrain from the frequent use of hearing protection devices because they do not see many benefits gained from these devices. This means that the demonstration of the benefit of the new technology would be helpful to enhance their understanding of how it can provide valuable information, such as safety acoustic cues. 

Lastly, human–computer interaction is worth investigating for future hearing assistance technologies for construction workers. Hazard detection results made by an audio processing technology would be useless if the involved parties did not trust it. Since construction sites, especially roadway work zones, are considerably harsh working environments due to complex noises generated from traffic and construction activities, errors such as false alarms or missing true alerts would be unavoidable even with the most advanced deep learning networks. Thus, human involved in hazard assessment is crucial to provide real-time feedback to enhance the decision makings. For example, advanced sound filtering that can suppress unwanted background noises to an appropriate signal/noise ratio before passing the environmental sound to the workers through hearing protection equipment would allow them to process the sound themselves, resulting in human computing teaming. To facilitate such collaboration, human-AI systems for audio signal processing should be appropriately designed considering workers’ cognition capabilities and other barriers regarding limited vision and hearing while performing construction tasks, often in the nighttime. 

## 6. Conclusions

Sound-based hazard detection for construction safety is a new and emerging area. This paper enhances the body knowledge by analyzing the current auditory signal processing using AI and its implication for construction safety, summarizing research trends and identifying promising future research directions. Previous research on sound sensing in construction has focused on the classification of equipment activities. There are still inadequate research efforts in areas such as sound-based measurement of hazardousness, detection of abnormal situations, and construction workers’ recognition of hazards at the construction site, shown by a steady trend of growing publications. AI-based signal processing for hazard assessment has great potential for improving the detection of hazards on the construction site. Deep learning methods such as CNN or AE using MFCC features have been shown to achieve impressive accuracy.

Further research that employs AI to enhance the worker’s auditory situational awareness still faces various technical and non-technical challenges. Technical challenges include hazard assessment and real-time data processing detection of abnormal situations without trained data. The current body of knowledge merits further research to precisely assess the hazardousness degree as well as the location of hazard events. Additionally, more studies, involving the survey of future users, are needed to understand the construction workers’ recognition and localization of hazards at the construction site, unsafe behaviors to auditory events, and important ergonomic and ecological factors to their auditory situational awareness. These new understandings will enable a successful deployment of a human-AI system for the auditory sensing of construction hazards. Lastly, more studies on the important factors of the implementation of AI-based hearing protection devices and sound-sensing technology are required to justify the value of AI-based sound surveillance towards construction safety and to promote the user’s adoption. 

## Figures and Tables

**Table 1:** Summary of accidents caused by construction equipment between 1990 and 2007 in USA, adopted from [36].

No.	Type of Equipment	Number of Fatality Cases	Percentage of Cases in Reverse Direction	Percentage of Cases without Reverse Alarms
1	Dump truck	173	91.1%	12.1%
2	Excavator/backhoe	50	53.3%	4%
3	Private vehicle (car, pickup, van)	42	28.6%	-
4	Dozer	38	82.4%	-
5	Grader	37	91.9%	13.5%
6	Front-end loader	31	72.4%	-
7	Forklift	30	57.1%	-
8	Tractor-trailer	24	54.5%	-
9	Compactor	18	82.4%	-
10	Scraper	15	80.0%	26.7%
11	Skid Steer Loader	15	92.9%	-
12	Water truck	13	91.7%	15.4%
13	Paver	8	-	-
14	Tractor (agricultural)	6	-	-
15	Crane	4	-	-
16	Sweeper	4	-	-

Note: empty cells indicate that the figures were not mentioned in the reference.

**Table 2:** Summary of factors causing the decline in auditory situational awareness of construction workers.

Factors			Conclusion	References
Jobsite-related	1	There are a significant number of sound sources in the acoustic event at construction sites	Even normal hearing listeners have a poor performance when complex auditory events happen	[38]
	2	Construction sites are considered noisy workplaces	Important sounds can be easily ignored or misidentified when there is the presence of extraneous noise	[39]
Worker-related	3	The majority of construction workers are male	Men have higher rates of difficulty in hearing than women	[3]
	4	Construction workers may experience hearing loss (due to hazardous noise)	Noise-exposed workers with hearing loss face many problems of job safety as the result of a reduction of hearing ability to warning signals	[3]

**Table 3:** Applications of sound surveillance.

Application	References
Home security	[23]
Detection of critical situations in a railway system	[24]
General surveillance in public areas	[16,17,19,20,21,22]
Detection of crimes in elevators	[25]
Detection of human emotions in public spaces	[18,26]
Office surveillance	[29]
Medical and health care facilities	
Surveillance of the elderly, the convalescent, or pregnant women	[27]
Automatic fall detection to improve the quality of life for independent older adults	[28]
Industrial plants	
Fault diagnosis of an induction motor	[31]
Detection of abnormality or failure of equipment	[32]

**Table 5:** Summary of traditional machine learning techniques for sound classification.

	Techniques	References
1	Gaussian Mixture Models (GMM)	[16,17,19,20,23,24,25,27,62,68,71]
2	Support Vector Machine (SVM)	[11,21,24,28,30,44,57,72,73,74]
3	K-Means Algorithms	[21,29,46,48,51]
4	Hidden Markov Models (HMM)	[22,45,47,63,75]
5	K-Nearest Neighbor Algorithms (KNN)	[31,57]

**Table 6:** Summary of deep learning techniques for sound classification.

Techniques	References
Deep Neural Network (DNN)	[58,59,60]
Convolutional Neural Network (CNN)	[9,12,28,46,70]
Recurrent Neural Network (RNN)/Bi-Directional Long Short-Term Memory (BLSTM)	[62,76]
Auto Encoder (AE)	[32,76,77]

**Table 7:** Summary of methods for sound localization.

Methods	References
1. Maximum-Likelihood Generalized Cross Correlation (GCC)	[17]
2. Linear-correction least-square localization algorithm	[17]
3. Similarity measure based on the Euclidean Distance (EUD) in the time domain	[78]
4. Similarity measure based on Normalized Cross Correlation (NCC) in the frequency domain	[79]
5. Similarity measure based on the Euclidean Distance (EUD) in the frequency domain	[80]
6. Fast Fourier Transform (FFT)	[80]
7. Fingerprinting algorithm	[81]

**Table 8:** Summary of auditory event characteristics in the construction field.

Code	Auditory Event Characteristics		References
A	Abnormality of sound		
	Greater than the threshold?		[71]
B	Direction of moving sound source		
	Towards the worker?		[78,79,80,81]
C	Location of sound		
	Less than a safe distance to the worker?		[78,79,80,81]
D	Type of sound		
D.1	Alert alarm (fire/earthquake)		[23,25,76,77]
D.2	Explosion		[19]
D.3	Gunshot		[16,17,19,20,25,61,62]
D.4	Human	a. Announcement	[23]
		b. Crowd ambient sound	[26]
		c. Scream/shout/cry	[17,18,19,20,23,24,25,26,27,28,61,62,68,76,77]
D.5	Moving heavy equipment/machine		[30,33,36]

**Table 9:** List of hazardous situations and required auditory event characteristics.

No.	Hazardous Situations	Combination of Auditory Event Characteristics
1	Heavy equipment/machine is at an unsafe distance	D.5 + C
2	Heavy equipment/machine is approaching	D.5 + C + B
3	Heavy equipment/machine is operating in an abnormal condition	D.5 + A
4	There is someone screaming/shouting/crying	D.4c
5	There is a crowd approaching	D.4b + B
6	There is an abnormal crowd approaching	D.4b + B + A
7	There is an alert alarm	D.1
8	There is an explosion	D.2
9	There is a gunshot	D.3
10	There is an abnormal sound	A

**Table 10:** Influencing factors of hearing protection devices.

No.	Influencing Factors	References
1	Planning effective interventions	[104]
2	Workplace interpersonal interaction	[105,107]
3	Poor insertion and poor fitting of earplugs	[106]
4	Left and right ear differences	[106]
5	Perception of noise	[107]
6	Hearing protection use	[107]
7	Reluctance to use HPDs	[107]
8	Value of hearing influence hearing protection use	[104,107]
