# Correction: Dang et al. Augmented Hearing of Auditory Safety Cues for Construction Workers: A Systematic Literature Review. *Sensors* 2022, *22*, 9135

**DOI:** 10.3390/s23229160

**Published:** 2023-11-14

**Authors:** Khang Dang, Kehinde Elelu, Tuyen Le, Chau Le

**Affiliations:** 1 Department of Informatics, Ying Wu College of Computing, New Jersey Institute of Technology, University Heights, Newark, NJ 07102, USA; kdangho@g.clemson.edu; 2 Glenn Department of Civil Engineering, College of Engineering, Computing and Applied Sciences, Clemson University, Lowry Hall, Clemson, SC 29634, USA; kelelu@g.clemson.edu; 3 Department of Civil, Construction and Environmental Engineering, College of Engineering, North Dakota State University, Fargo, ND 58102, USA; chau.le@ndsu.edu

## 1. Error in Table

In the original publication [1], there was an error in the published Table 9: “List of hazardous situations and required auditory event characteristics (see Table 8 for the auditory event notations)”. There were several incorrectly typed words in the last column of Table 9. The corrected Table 9 is presented below.

## 2. Addition of an Author

Khang Dang was not included as an author in the original publication. The corrected Author Contributions statement appears here.

**Author Contributions:** Conceptualization, K.D. and T.L.; investigation, K.D. (reviewed 78 articles), K.E. (reviewed 7 articles) and T.L.; methodology, K.D.; visualization, K.D.; supervision, T.L.; writing—original draft, K.D. (Sections 1, 2, 3, 4.1, 4.2.1 and 6) and K.E. (Sections 4.2.2 and 5); writing—review and editing, K.D., K.E., T.L. and C.L. All authors have read and agreed to the published version of the manuscript.

The authors state that the scientific conclusions are unaffected. This correction was approved by the Academic Editor. The original publication has also been updated.

## Figures and Tables

**Table 9:** List of hazardous situations and required auditory event characteristics.

No.	Hazardous Situations	Combination of Auditory Event Characteristics
1	Heavy equipment/machine is at an unsafe distance	D.5 + C
2	Heavy equipment/machine is approaching	D.5 + C + B
3	Heavy equipment/machine is operating in an abnormal condition	D.5 + A
4	There is someone screaming/shouting/crying	D.4c
5	There is a crowd approaching	D.4b + B
6	There is an abnormal crowd approaching	D.4b + B + A
7	There is an alert alarm	D.1
8	There is an explosion	D.2
9	There is a gunshot	D.3
10	There is an abnormal sound	A

## References

[1] Dang K., Elelu K., Le T., Le C. (2022). Augmented Hearing of Auditory Safety Cues for Construction Workers: A Systematic Literature Review. Sensors.

